# A novel tissue-selective β2-adrenoceptor agonist with minimized cardiovascular effects, 5-HOB, attenuates neuropathic pain in mice

**DOI:** 10.1186/s13104-019-4466-y

**Published:** 2019-07-15

**Authors:** Marie Jourdain, Shinji Hatakeyama

**Affiliations:** Novartis Institutes for BioMedical Research, Novartis Pharma AG, Basel, Switzerland

**Keywords:** Neuropathic pain, Allodynia, β2 adrenoceptor agonist, 5-HOB

## Abstract

**Objective:**

5-HOB is a novel tissue selective, 5-hydroxybenzothiazolone-derived β2 adrenoceptor agonist with minimized cardiovascular effects while retaining efficacy on skeletal muscle in preclinical experiments unlike conventional β2 adrenoceptor agonists, however its effect on the nervous system has not been evaluated yet. Therefore, 5-HOB was evaluated in a mouse model of neuropathic pain.

**Results:**

5-HOB alleviated neuropathic allodynia in a dose dependent manner and reversed the changes in hind paw withdrawal thresholds to the sham control levels. The dose attenuating neuropathic allodynia was slightly lower than the dose inducing skeletal muscle hypertrophy. In conclusion, as reported with known β2 adrenoceptor agonists, 5-HOB was also effective in attenuating neuropathic pain in mice in addition to its effect on skeletal muscle.

## Introduction

Neuropathic pain is a major type of chronic pain, which is initiated by a lesion in the sensory neuron due to e.g. trauma, postsurgical injuries, anticancer drugs, or by a disease directly affecting the somatosensory system, such as diabetes, multiple sclerosis or cancer [1]. Severe symptoms associating with neuropathic pain, such as allodynia, aching, tingling, numbness, burning, cramp-like pain, stabbing, and shock-like pain, can have a significant negative impact on the quality of life and the activities of daily living of the patients. Clinically, tricyclic antidepressants and serotonin-norepinephrine reuptake inhibitors are recommended as first-line therapies [2]. The clinical condition does not always correlate well with the status of a causative disease in many cases and often persists even after the causative disease is cured [2]. Despite its high prevalence, the treatment of neuropathic pain is often challenging due to central nervous system side effects with first-line therapies. Therefore, there is still a high-unmet medical need for a better treatment of neuropathic pain compared the currently available therapies.

In the nociceptive system, β2 adrenoceptor is expressed and essential for the anti-neuropathic pain effect of antidepressants such as tricyclic antidepressants and serotonin-norepinephrine reuptake inhibitors [3, 4]. β2 adrenoceptor agonists are also capable of attenuating allodynia after chronic treatment as effectively as anti-depressants [5, 6], improving hyperalgesia and electrophysiological nerve dysfunction in diabetic rodents [7, 8], and promoting regeneration after nerve injury [9]. Moreover, in an epidemiological study on patients operated by thoracotomy, the chronic use of β2 adrenoceptor agonists was associated with a five-fold decrease in the relative incidence of neuropathic pain [10].

5-HOB is a novel tissue selective, 5-hydroxybenzothiazolone-derived β2 adrenoceptor agonist with minimized cardiovascular effects while retaining efficacy on skeletal muscle in preclinical experiments unlike conventional β2 adrenoceptor agonists [11], however its effect on the nervous system has not been evaluated yet. Therefore, 5-HOB was evaluated in a mouse model of neuropathic pain [12].

## Main text

### Methods

#### Animals and maintenance conditions

The experiment described in this report was performed according to the regulations effective in the Canton of Basel-City, Switzerland, under the license number BS-2489. C57BL/6JRj male mice at the age of 11 weeks were purchased from Janvier Labs (Le Genest-Saint-Isle, France). The mice were housed at 25 °C with a 12:12 h light–dark cycle with specific pathogen free condition in a group of 2 to 4 and acclimated to the facility for 7 days, but some aggressive or incompatible mice were isolated and housed individually. Food and water were provided ad libitum. They were fed a standard laboratory diet containing 18.2% protein and 3.0% fat with an energy content of 15.8 MJ/kg (Kliba Nafag 3890, Provimi Kliba AG, Kaiseraugst, Switzerland).

#### Materials

5-HOB (salt factor = 1.139) was synthesized in Technical Research and Development of Novartis Pharma AG, and 5-HOB was dissolved in saline, and the doses were quoted as the non-salt.

#### Chronic constriction injury (CCI)

CCI was performed to induce neuropathic allodynia as described [12]. Polyethylene tubing PE-20 (inner/outer diameter 0.38/1.09 mm) was cut into segments of 2 mm in length. Cuffs were then slit longitudinally along one side so that they could be opened with fine forceps and placed around the nerve. Once prepared, cuffs were stored in a Petri dish filled with 70% ethanol until use. Mice were treated subcutaneously with carprofen at a dose of 5 mg/kg and warm lactated Ringer solution (10 mL/kg) about 0.5 to 1 h prior to surgery, and anesthetized using isoflurane at a concentration of 3 to 5% for induction and 1 to 3% for maintenance. Under aseptic conditions, the hind limb of an anesthetized mouse was shaved and the sciatic nerve was isolated. Then, a polyethylene cuff was placed around the sciatic nerve, and the nerve was crushed through the cuff with forceps, 3 times for 1 s. The wound was closed with clips, and after the surgery the mice were treated with carprofen once per day for 2 to 3 days depending on the condition of the mouse. The clips were removed 10 days later.

#### Nociceptive test

CCI-induced mechanical allodynia was evaluated by measuring the mechanical threshold of hind-paw withdrawal using von Frey hairs as described [12]. The mice were first placed in the procedure room during 45 min for a first habituation, and then placed in clear Plexiglas boxes on an elevated mesh screen (Bioseb, Vitrolles, France), and allowed to habituate again for 15 min before testing. The calibrated von Frey filaments (Aesthesio: Bioseb, Vitrolles, France) were applied to the plantar surface of each hind paw in a series of ascending forces, and each filament was evaluated five times per paw. The threshold was defined as the force that resulted in three or more hind paw withdrawals in five attempts.

#### Experimental design

A dose–response effect of 5-HOB on CCI-induced allodynia was evaluated, and the treatment was initiated 14 days after surgery, when allodynia was established and stable. After 2 to 3 habituation rounds in the nociceptive test, the baseline values before surgery (day-1) were collected, and the mice for the sham vehicle group were separated. The values collected on day 13 were used for randomization of the mice with CCI prior to the start of treatment on day 14, and then the mechanical threshold was measured twice weekly. Saline was administered to the Sham and CCI vehicle controls. 5-HOB was administered subcutaneously at 0.001, 0.003, 0.01 and 0.03 mg/kg in a volume of 5 mL/kg, twice daily on weekdays and once daily on weekends; n = 8 in all groups except n = 7 in the CCI group treated with 5-HOB at 0.03 mg/kg. The mice were euthanized with CO_2_ on day 42.

Values are expressed as mean ± SEM. Statistical analysis was carried out to compare the treatment groups to Sham Vehicle or CCI Vehicle, using Tukey’s multiple comparison test following 2 way ANOVA for the time course analysis, and Sidak’s multiple comparison test following 1 way ANOVA for the analysis on day 42. Differences were considered to be significant when the probability value was < 0.05. Statistical analyses were performed by GraphPad Prism (GraphPad Software, La Jolla, CA, USA).

### Results and discussion

**Fig. 1:**
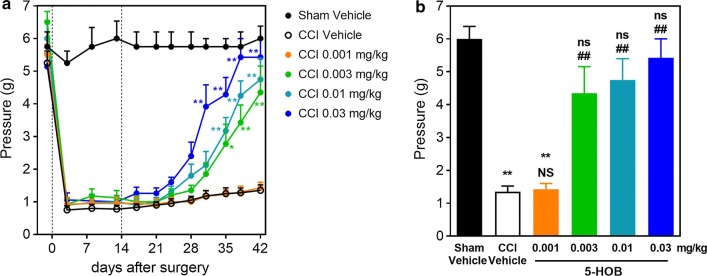
Effect of 5-HOB on neuropathic allodynia (CCI operated). Effect of 5-HOB on the operated hind paw withdrawal threshold to pressure of von Frey hairs in time course (**a**) and on day 42 (**b**). The dotted lines in A indicate the surgery performed on day 0 and the treatment start on day 14 with vehicle or 5-HOB. Values are expressed as means ± SEM (n = 7 ~ 8). **a** Tukey’s multiple comparison test following 2 way ANOVA; ^*^P < 0.05, ^**^P < 0.01 versus CCI Vehicle. **b** Sidak’s multiple comparison test following 1 way ANOVA; ^**^P < 0.01, ns: no significant difference versus Sham Vehicle, ^##^*P* < 0.01, NS: no significant difference versus CCI Vehicle

**Fig. 2:**
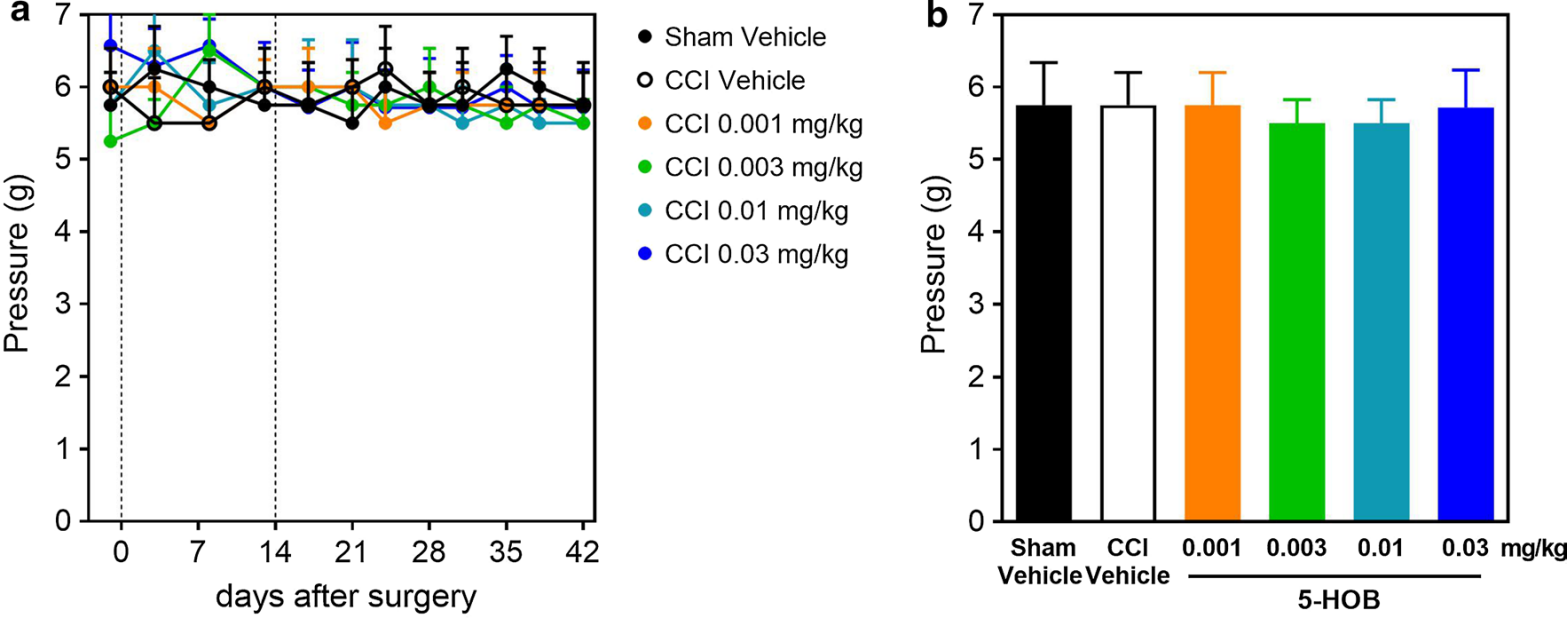
Effect of 5-HOB on neuropathic allodynia (contralateral). Effect of 5-HOB on the contralateral non-operated hind paw withdrawal threshold to pressure of von Frey hairs in time course (**a**) and on day 42 (**b**). The dotted lines in A indicate the surgery performed on day 0 and the treatment start on day 14 with vehicle or 5-HOB. Values are expressed as means ± SEM (n = 7 ~ 8). No significant differences when compared to Sham Vehicle and CCI Vehicle

A dose–response effect of 5-HOB on allodynia was evaluated in a mouse model of neuropathic pain to define an efficacious dose. Chronic constriction injury (CCI) was performed on day 0 to induce allodynia, and the treatment was initiated on day 14 when allodynia was established and stable. At higher doses, the anti-allodynia effect of 5-HOB emerged around day 28, reaching statistical significance on day 31 at 0.03 mg/kg when compared to CCI vehicle control, and day 35 for all doses except the lowest dose of 0.001 mg/kg (Fig. 1a). On day 42, 5-HOB dose dependently reversed the withdrawal threshold to pressure of von Frey hairs close to Sham vehicle control level, and the tolerated pressure was not statistically significant from Sham vehicle control except for the lowest dose (Fig. 1b). Therefore, the lowest efficacious dose was defined as 0.003 mg/kg in this experiment. On the other hand, in the contralateral paws, CCI vehicle control and 5-HOB treatment showed no significant effects on hind paw withdrawal threshold (Fig. 2a, b).

In this report, a selective β2 adrenoceptor agonist 5-HOB was evaluated in a mouse model of neuropathic pain to define an efficacious dose. 5-HOB was administered subcutaneously twice daily on weekdays and once daily on weekends. In this model, 5-HOB alleviated neuropathic allodynia in a dose dependent manner and reversed the hind paw withdrawal threshold to the Sham vehicle control level, with the minimum efficacious dose of 0.003 mg/kg. The dose attenuating neuropathic allodynia was slightly lower than the dose inducing skeletal muscle hypertrophy [11]. Since patients with peripheral neuropathy often show muscle weakness, a dual effect of 5-HOB on allodynia and skeletal muscle might be beneficial to such patients suffering from neuropathic pain and muscle weakness [13]. In conclusion, as reported with known β2 adrenoceptor agonists, 5-HOB was also effective in attenuating neuropathic pain in mice in addition to its effect on skeletal muscle.

## Limitations

In the experiment described in this report, we only evaluated the efficacy of 5-HOB in the CCI model and did not assess any biomarkers related to neuropathic pain, neuronal degeneration and regeneration after CCI, and β2 adrenoceptor pathway. Further assessment in various biomarkers as well as additional neuropathic pain models such as diabetes or chemotherapy-induced neuropathic pain will be required to validate the effect of 5-HOB in neuropathic pain.

## Data Availability

The dataset used in the current experiment is available from the corresponding author by request.

## Abbreviations

5-HOB: 5-hydroxybenzothiazolone
CCI: chronic constriction injury
